# An overlook on the current registries for rare and complex connective tissue diseases and the future scenario of TogethERN ReCONNET

**DOI:** 10.3389/fmed.2022.889997

**Published:** 2022-09-26

**Authors:** Matilde Bandeira, Federica Di Cianni, Diana Marinello, Laurent Arnaud, Sara Cannizzo, Claudio Carta, Alain Cornet, Sara M. Barril, Inita Bulina, Alessandro Ferraris, João Fonseca, Andrea Gaglioti, Marteen Limper, Valentina Lorenzoni, Judith Majnik, Marco Matucci-Cerinic, Ilaria Palla, Simona Rednic, Matthias Schneider, Vanessa Smith, Alberto Sulli, Klaus Søndergaard, Simone Ticciati, Angela Tincani, Giuseppe Turchetti, Rosaria Talarico, Maurizio Cutolo, Marta Mosca, Domenica Taruscio

**Affiliations:** ^1^Rheumatology Department, Lisbon Academic Medical Centre, Hospital de Santa Maria, Centro Hospitalar Universitário Lisboa Norte, Lisbon, Portugal; ^2^Rheumatology Research Unit, Instituto de Medicina Molecular João Lobo Antunes, Faculdade de Medicina, Universidade de Lisboa, Lisbon, Portugal; ^3^Rheumatology Unit, Azienda Ospedaliero Universitaria Pisana, University of Pisa, Pisa, Italy; ^4^Rheumatology Department, Hôpitaux Universitaires de Strasbourg, Centre National de Référence des Maladies Systémiques et Auto-immunes Rares Grand-Est Sud-Ouest, Strasbourg, France; ^5^Institute of Management, Sant'Anna School of Advanced Studies, Pisa, Italy; ^6^National Centre for Rare Diseases, Istituto Superiore di Sanità, Rome, Italy; ^7^Lupus Europe, Brussels, Belgium; ^8^Rheumatology Department, Hospital Universitari Vall d'Hebron, Barcelona, Spain; ^9^Department of Rheumatology, Stradins Clinical University Hospital, Riga, Latvia; ^10^Medical Genetics Laboratory, Molecular Medicine Department, San Camillo Forlanini Hospital, Sapienza University, Rome, Italy; ^11^Department of Rheumatology and Clinical Immunology, University Medical Center Utrecht, Utrecht University, Utrecht, Netherlands; ^12^Department of Rheumatology and Clinical Immunology, Semmelweis University, Budapest, Hungary; ^13^Division of Rheumatology and Scleroderma Unit, Department of Clinical and Experimental Medicine, Azienda Ospedaliero Universitaria (AOU) Careggi, University of Florence, Florence, Italy; ^14^Unit of Immunology, Rheumatology, Allergy and Rare Diseases, IRCCS San Raffaele Hospital, Milan, Italy; ^15^Department of Rheumatology, Emergency County Teaching Hospital, University of Medicine and Pharmacy Iuliu Hatieganu, Cluj-Napoca, Romania; ^16^Department of Rheumatology, University Hospital Düsseldorf, Heinrich-Heine-University Düsseldorf, Düsseldorf, Germany; ^17^Department of Rheumatology, Department of Internal Medicine, Ghent University Hospital, Ghent, Belgium; ^18^Unit for Molecular Immunology and Inflammation, VIB Inflammation Research Centre, Ghent, Belgium; ^19^Laboratory of Experimental Rheumatology, Division of Clinical Rheumatology, Department of Internal Medicine (DIMI), University of Genoa, Genoa, Italy; ^20^IRCCS Polyclinic Hospital San Martino, Genoa, Italy; ^21^Department of Rheumatology, Aarhus University Hospital, Aarhus, Denmark; ^22^Rheumatology and Clinical Immunology Unit, ASST-Spedali Civili, University of Brescia, Brescia, Italy

**Keywords:** rare and complex connective tissue diseases, registries, ERN ReCONNET, TogethERN ReCONNET, European Reference Networks

## Abstract

**Background:**

Patient registries play a crucial role in supporting clinical practice, healthcare planning and medical research, offering a real-world picture on rare and complex connective tissue diseases (rCTDs). ERN ReCONNET launched the first European Registry Infrastructure with the aim to plan, upgrade and link registries for rCTDs, with the final goal to promote a harmonized data collection approach all over Europe for rCTDs.

**Methods:**

An online survey addressed to healthcare professionals and patients' representatives active in the field of rCTDs was integrated by an extensive database search in order to build a mapping of existing registries for rCTDs.

**Findings:**

A total of 140 registries were found, 38 of which include multiple diseases. No disease-specific registry was identified for relapsing polychondritis, mixed connective tissue disease and undifferentiated connective tissue disease.

**Discussion:**

This overview on the existing registries for rCTDs provides a useful starting point to identify the gaps and the strengths of registries on the coverage of rCTDs, and to develop a common data set and data collection approach for the establishment of the TogethERN ReCONNET Infrastructure.

## Key messages

- Registries produce a real-world picture of diseases in all their aspects and may play a crucial role in supporting health care professionals in clinical practice and healthcare planning. Therefore, registries and databases constitute a key opportunity to enhance further medical research in a very challenging field.
- Recently, the ERN ReCONNET launched the first European Registry Infrastructure (TogethERN ReCONNET) with the aim to plan, upgrade and link registries for rare and complex connective tissue diseases (rCTDs), with the final goal to promote a harmonized data collection approach all over Europe for rCTDs.
- The present work, conducted in the framework of the ERN ReCONNET, was aimed at performing a mapping of all the existing registries for rCTDs and it was done by an extensive and systematic search. A total of 140 registries were found, 38 of which include multiple diseases. No disease-specific registry was identified for relapsing polychondritis, mixed connective tissue disease and undifferentiated connective tissue disease.
- This overview provides the starting point to identify the gaps and the strengths of registries on the coverage of rCTDs, and to develop a common data set and data collection approach for the establishment of the TogethERN ReCONNET Infrastructure.
- Collecting more evidence on some neglected rCTDs might surely have a big impact not only in clinical practice but also in the future health planning processes of policy makers.

## Background

Rare connective tissue and musculoskeletal diseases (rCTDs), along with all the rare and complex conditions affecting the lives of millions of European citizens, represent a big challenge for European Union's (EU) health systems in the attempt to provide high-quality and homogeneous care. In line with this, the ERN ReCONNET (European Reference Network on Rare and Complex Connective Tissue and Musculoskeletal Diseases) (1) is one of the 24 ERNs (2) established by the European Commission as virtual networks involving health care professionals, hospitals and patients' organizations across Europe to promote and facilitate the discussion on rare and complex diseases, offering equal access to the best possible health care and exchange of knowledge. ERN ReCONNET was established with the purpose to improve the management of rCTDs across EU, bringing together rCTDs patients' representatives and the leading European centers with expertise in diagnosis and treatment of these diseases.

At the time of the finalization of the manuscript, the ERN ReCONNET involved 38 health care providers (HCPs) from eighteen different European countries: Austria, Belgium, Croatia, Denmark, Estonia, France, Germany, Hungary, Italy, Latvia, Lithuania, Luxembourg, Malta, Netherlands, Portugal, Romania, Spain and Slovenia; moreover, as of 1st January, 30 new members HCPs have joined the ERN ReCONNET as full members and therefore they will join the next activities of the TogethERN ReCONNET. The Network covers 10 rare and complex connective tissue diseases: systemic sclerosis (SSc), mixed connective tissue diseases (MCTD), idiopathic inflammatory myopathies (IIM), antiphospholipid syndrome (APS), undifferentiated connective tissue diseases (UCTD), IgG4-related diseases (IgG4), relapsing polychondritis (RP), systemic lupus erythematosus (SLE), Sjögren's syndrome (SS), Ehlers- Danlos syndromes (EDS). The main goals of ERN ReCONNET include not only the increase of knowledge and the improvement of the management of rCTDs, but also the facilitation of data sharing and harmonization of data collection across borders.

To accomplish these goals, special support to research initiatives is needed, due to the low number of patients and high phenotype diversity of rCTDs. In fact, evidence published to date offers precious but still incomplete understanding of these conditions in terms of natural history, manifestations and outcomes, revealing how clinical research in this field remains challenging. Part of this challenge can be efficiently addressed through a systematic collection of clinical, epidemiological, genetic and biologic data in the form of patient registries. As defined by the European Medicine Agency “*Patient registries are organised systems that use observational methods to collect uniform data on a population defined by a particular disease, condition or exposure, and that is followed over time*”. Patient registries may represent a crucial tool to observe the course of disease, the variables that influence the prognosis and the effectiveness of treatment options, and they facilitate multidisciplinary collaboration with the overall aim of improving the overall patients' quality of care. Registries produce a real-world picture of diseases in all their aspects and may play a crucial role in supporting health care professionals in clinical practice and healthcare planning. However, clinicians are not the only stakeholders who may benefit from the value of registries. For example, for physicians' organizations, a registry might provide data that can be used to assess if clinicians are managing a disease in accordance with evidence-based guidelines. Moreover, registries may also play an important social role as well, by connecting patients and families who are facing similar challenges as well as clinicians working in the same disease area.

Above all else, registries and databases constitute a key opportunity to enhance further medical research in the field of rare diseases. Indeed, they offer the ideal recruitment platform to launch studies with an adequate sample size and they also provide outcome results that may be generalizable for a wide range of patients, as data collected reflect real-world experience (3, 4).

In many European countries clinicians collect patients' data for rCTDs in hundreds of local and regional/national registries, as part of their routine clinical practice. As stated before, these heterogeneous and fragmented data are likely to complement each other and to provide a unique opportunity to better understand epidemiological and clinical features of these conditions.

For this reason, the European Commission decided to set up a European Platform on Rare Disease Registration (EU RD Platform), to promote the interoperability of data in rare diseases registries, providing the standards and the tools on an EU-level for data collection and exchange in rare diseases covered by each ERN. Besides, the European Commission has also launched a call to support the development of rare disease (RD) registries for the ERNs, and in 2020 ERN ReCONNET launched the first European Registry Infrastructure for rCTDs, called TogethERN ReCONNET, with the aim to plan, upgrade and link registries of rCTDs. The final goal of TogethERN is to promote a harmonized data collection approach of rCTDs all over Europe.

The first activity of TogethERN ReCONNET was the creation of a working group consisting of expert clinicians, researchers, fellows of the network and patients' representatives, to co- design and perform a mapping of the existing registries of rCTDs. Accordingly, this paper aims to outline the current scenario of rCTDs registries across Europe, in an attempt to provide a starting point for the development of TogethERN Infrastructure.

## Methods

The study was co-designed with the TogethERN ReCONNET Working Group dedicated to this task. The study included a total of 3 phases.

In Phase 1, an on-line anonymous survey was co-designed with the support of the Working Group. The main objective of the survey was to identify any existing registries, exploring the profile of the respondent (age, country, involvement in ERN ReCONNET, etc.) and the awareness of the respondents of any existing rCTDs registry. Different details of the registries were also asked for, e.g., the disease(s) covered by the registry, the geographical coverage, collection of the minimum data set for rare diseases developed by the Joint Research Centre, etc. (5, 6). The Survey was uploaded in the EU Survey Platform (7) and launched across the rCTDs community of healthcare professionals and patients' organizations both *via* email and *via* social media (Facebook and Twitter).

In Phase 2, two fellows belonging to the centers involved in the Working Group performed an extensive research on other registries that were not reported by the survey. The references for this review were identified through searches in PubMed (8), Orphanet (9) and RDConnect (10, 11). Registries were found using the search terms “registry” and the name of each specific disease from the ERN ReCONNET rare and complex connective tissue diseases list. Additional searches were conducted to find additional data on registries found within websites and informal sources.

**Figure 1:**
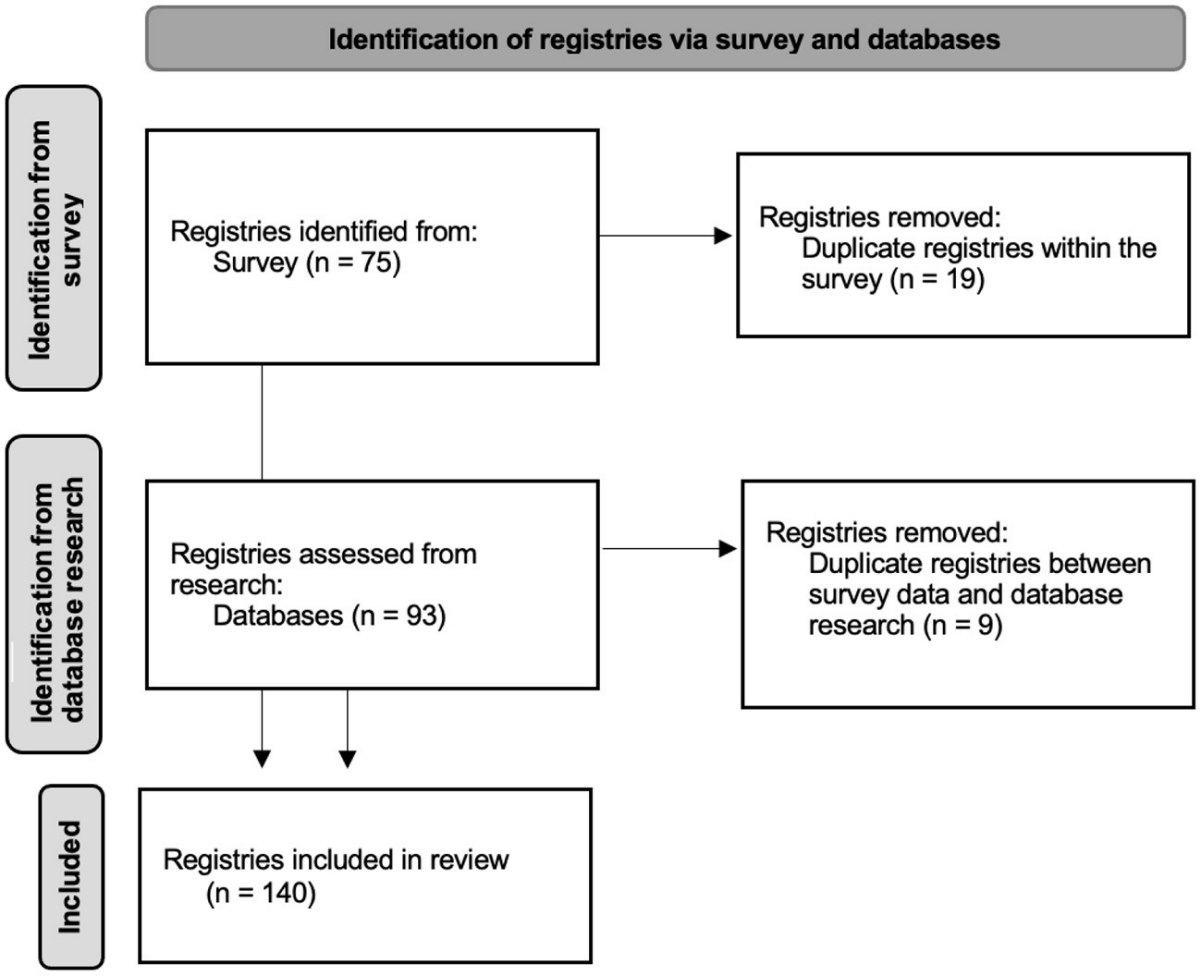
PRISMA chart displaying the identification of the registries.

The third phase of the study included the analysis of the survey results as well as the incorporation of the results identified in Phase 2, to obtain the final list of rCTDs registries identified. Registries found on the survey were complemented by the registries uncovered by the database research according to Figure 1. Descriptive data were reported.

## Results

The online survey was sent *via* email to all ERN ReCONNET HCPs, European Patient Advocacy Groups (ePAGs) and rCTDs patients organizations. The survey was also disseminated *via* social networks to encompass more patients and other clinical experts and avoid under-reporting of local, less diffused, registries. The survey was launched on 5th of July and closed on the 31st of August of 2021.

The survey consisted of 4 multiple choice questions with an open question needed to specify details, 11 yes or no questions, 4 open questions, 2 of which were optional, and 9 fields for each registry the participant mentioned. In summary, respondents were asked how they were affiliated to ERN ReCONNET, which country they were from and then were asked to describe registries in which they were involved whether as a coordinator, participant or simply aware of its existence. Data about name, website, date of launch, disease scope and geographic coverage of each registry were collected. Participants were asked to fill in information regarding reference publications or reports/websites in which the registry was mentioned or related to work done through that registry's data.

**Figure 2:**
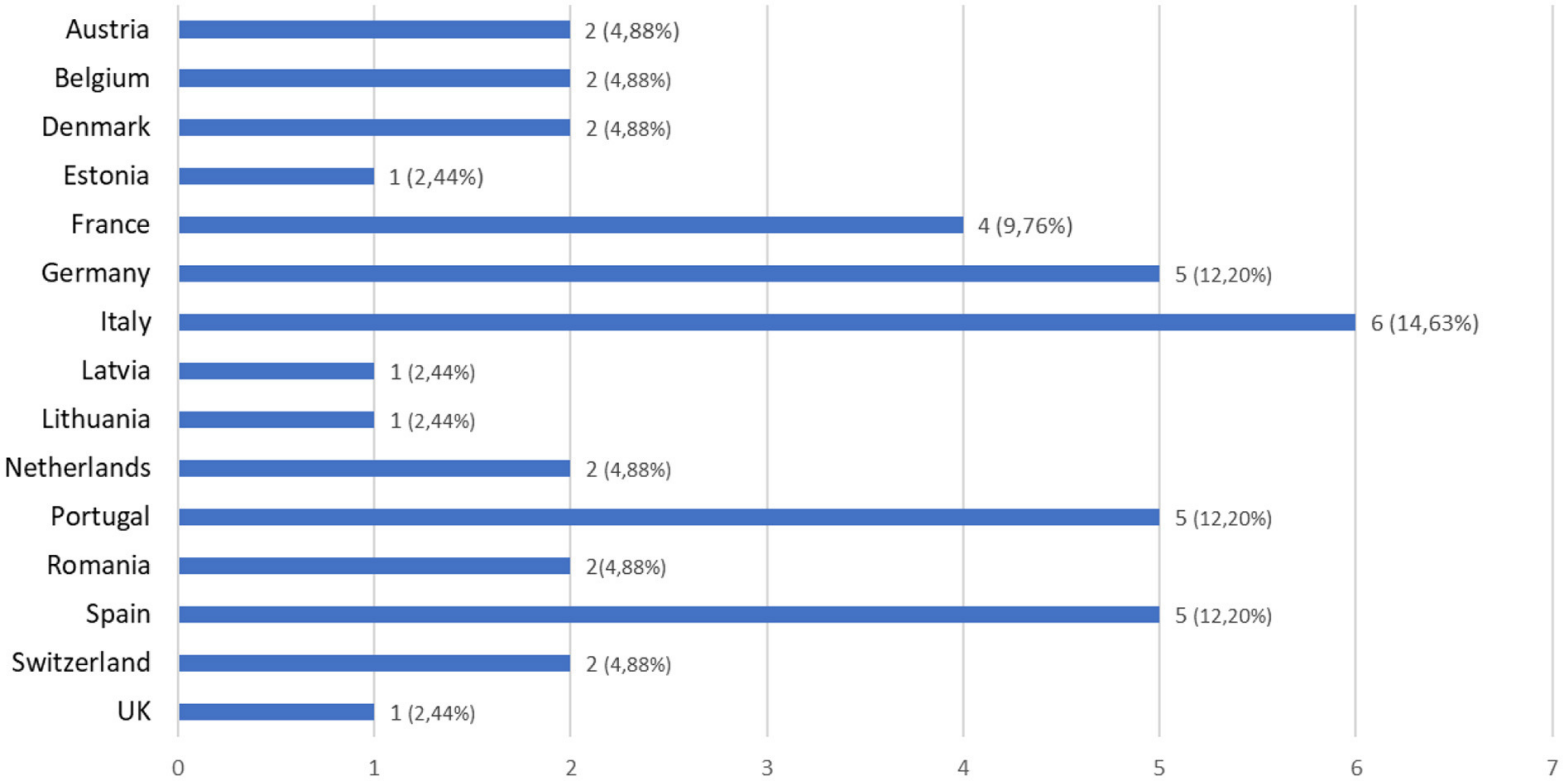
Country of respondents to the survey (*n* = 41).

The total number of replies was 41, with replies from 15 different European countries (Figure 2), a majority from Italy (14.63%), Spain (12.20%), Portugal (12.20%), Germany (12.20%) and France (9.76%).

**Figure 3:**
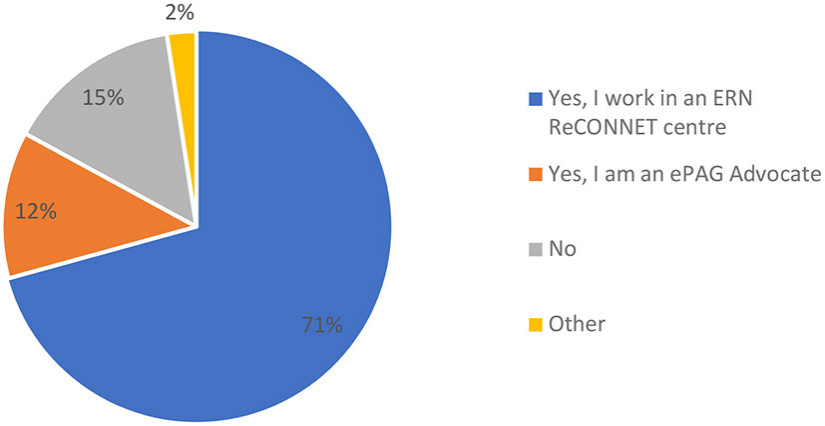
Affiliations of participants of the survey to ERN ReCONNET (*n* = 41).

In terms of the profile of respondents, clinicians, researchers or other types of experts on rCTDs gave most replies (73%) followed by patients and patient representatives (22%). Most respondents (83%) were directly involved in ERN ReCONNET, either as an expert of an ERN ReCONNET center (71%) or as an ePAG advocate (12%), with 15% of the participants unaffiliated to ERN ReCONNET (Figure 3).

A total of 140 registries were found as showed in Figure 1. No registries were excluded except for the duplicates. It was this study's goal to have wide inclusion criteria to include local/regional less well-known registries that are otherwise ignored in most of the literature. Registries outside the scope of Europe were also included. Of the 140 registries found, 38 include multiple diseases (Supplementary Table 1). Some registries were specifically dedicated to ERN ReCONNET diseases: IIM (28 registries); SSc (19), EDS (17); SLE (14); SS (10); APS had; IgG4-related diseases (5). Disease-specific registries for RP, MCTD, and UCTD were not found, although these diseases are included in some multiple-disease registries.

### Specific-disease registries for IIM

Registries for IIM include 2 registries for juvenile myopathies (see rows 33, 37 in Supplementary Table 1), 2 registries that specifically encompass both adult and juvenile myopathies (see rows 32, 34 in Supplementary Table 1), one registry for clinically amyopathic dermatomyositis (see rows 55 in Supplementary Table 1), one registry for inclusion body myositis (see row 56 in Supplementary Table 1) registries for antisynthetase syndrome (see rows 44, 48, 49 in Supplementary Table 1). The most of registries on IIM are multicentre, while 9 are international (see rows 32, 34, 35, 37, 38, 48, 49, 52, 56 in Supplementary Table 1). Six of the registries included have a geographic coverage within areas exclusively outside of Europe (see rows 39, 40, 45, 47–59 in Supplementary Table 1).

### Specific-disease registries for SSc

There are registries that are dedicated to particular SSc manifestations/complications, for example pulmonary hypertension (see row 101 in Supplementary Table 1), digital ulcers (see row 99 in Supplementary Table 1) or morphea (see row 102 in Supplementary Table 1). Most registries on SSc (9 out of 16) are either from Australia or from the American continent (see rows 87–89, 94, 97, 98, 100–102 in Supplementary Table 1). There are a few nationwide registries within Europe, as well as an international European ongoing registry (EUSTAR) (see row 86 in Supplementary Table 1).

### Specific-disease registries for SLE

SLE registries include 3 international (see rows 64, 65,72 in Supplementary Table 1) and 10 national registries, namely German, Spanish, American, Greek, Dutch, Italian, Romanian, Danish and Swiss (see rows 60, 62, 63, 66–71, 73 in Supplementary Table 1). There is a registry for pediatric and neonatal SLE (see row 60 in Supplementary Table 1) in Germany. It is also worth mentioning that various multiple disease registries also include SLE.

### Specific-disease registries for SS

SS available registries are mostly national/local registries and there seems to exist only one international registry ongoing, the Big Data Sjögren Consortium (see row 75 in Supplementary Table 1). There was no Sjögren Syndrome registry from outside of Europe found, with some multiple disease registries also including this disease.

### Specific-disease registries for APS

Of the 9 registries on APS, 4 are international (see rows 2–4, 7 in Supplementary Table 1) registries and 3 are nationals (see rows 1, 5, 8 in Supplementary Table 1), one of which has already been discontinued (see row 1 in Supplementary Table 1). Some were designed for a specific sub-population of patients with one for catastrophic APS (see row 3 in Supplementary Table 1) and another one for APS in pediatric age (see row 2 in Supplementary Table 1), as well as a recent one that focuses on APS and COVID-19 (see row 6 in Supplementary Table 1). One of the registries included all individuals with persistently positive antiphospholipid antibodies and not only APS patients (see row 4 in Supplementary Table 1). APS is also a recurrent disease included in multiple disease registries, especially in registries including SLE and IgG4-related disease.

### Specific-disease registries for EDS

Most registries on Ehlers-Danlos syndromes appear to be international (four (see rows 15, 17, 22, 23 in Supplementary Table 1) out of the total 17), with 5 national registries (see rows 10–12, 20, 24 in Supplementary Table 1) and one regional registry from the Netherlands (see row 21 in Supplementary Table 1). Within the EDS registries, 5 included biobank collections (see rows 14, 17–19, 21 in Supplementary Table 1) and another one included genetic data (see row 25 in Supplementary Table 1). There seems to be a significant number of registries within the scope of neuromuscular diseases that include EDS and were included in this study in the multiple disease registries.

### Specific-disease registries for IgG4-related disease

IgG4-related disease registries found were mainly national registries, one from France (see row 28 in Supplementary Table 1) that had been functioning up until 2016 and seemed to be substituted by a different French registry that started in 2019 (see row 31 in Supplementary Table 1), and 2 ongoing from Germany (see row 27 in Supplementary Table 1) and Spain (see row 29 in Supplementary Table 1). The only ongoing European registry started in 2010 and has recruited almost 500 patients (see row 30 in Supplementary Table 1).

## Discussion

This work shows that several registries are currently available for some rCTDs across Europe, yet still significant gaps were identified, specifically in the coverage of diseases with the lowest prevalence. At present, no disease-specific registry was identified for UCTD, MCTD and RP, likely reflecting the lower awareness and research activities concerning these rare conditions.

On the other hand, most of the existing registries reported in our mapping exercise cover multiple connective tissue diseases. This trend might reflect the growing need of physicians and researchers to connect, share and integrate data on conditions that belong to a common field, with the purpose of enhancing research and build a comprehensive knowledge base on these rare and complex diseases. In fact, TogethERN ReCONNET aims to meet this need by offering the tools and setting the standards to promote a synergic cooperation between networks and their registries.

While several sources are defending that international registries should be the gold standard, only a limited number of disease-specific registries included in this overview are international. In addition, many registries from extra-EU countries (North America, Australia, Korea, etc.) were included, covering primarily IIM, SSc and multiple diseases. However, the presence of local and national registries for a rare connective tissue disease is a valuable resource as it might pave the way toward the development of a common dataset for a further international registry or collaborative publications in a specific area, which can be facilitated by the TogethERN Infrastructure (12).

Moreover, it is important to notice that most of the registries identified and reported in this publication collect epidemiological and clinical data, making them adequate tools for assessing epidemiology and disease course of rCTDs across Europe. On the other hand, TogethERN infrastructure is expected to fill the gaps of those diseases not covered by any specific-disease registry, also with the aim to reach appropriate evidence on epidemiology and clinical course of the rarest connective tissue diseases. Research in this field is enhanced when data from patient registries are integrated with other data, such as biologic and imaging information, looking for biomarkers. In fact, biomarkers are important components of rCTDs, since they may be related to risk, exposure, prognosis, prediction of treatment outcome and etiological mechanisms. Unfortunately, very few biomarkers are available for rCTDs, so the existing registries for these conditions should aim at collecting biological samples (3, 13). In this regard, twenty registries were included in our mapping of rCTDs registries classified also as biobanks.

Some final interesting observations come from the methodology of this work. First, the results of the survey were integrated by additional research of rCTDs registries performed in the existing databases online, thus capturing registries that would have likely been missed otherwise. Secondly, it should be underlined that the quality of the identified registries was not addressed by this mapping activity. In fact, all the registries identified by the survey and the web-searching were included, regardless of the data setting, sample size, geographical coverage and current state (still ongoing/not ongoing).

This overview aims to give a starting point to identify the gaps and the strengths of registries covering rCTDs, which TogethERN ReCONNET is expected to fill and enhance, respectively. The biggest challenge is the current lack of registries for some rare clinical conditions. However, existing registries offer the basis to develop a common data set for TogethERN.

ReCONNET, in order to promote a homogeneous approach for data collection on rCTDs, by avoiding fragmentation and duplication of data. In addition, the future work of TogethERN ReCONNET will also include the alignment to the to the quality and Fairification requirements and to the European Platform on Rare Disease Registration, such as the adoption of the set of common data elements for Rare Diseases Registration.

## Conclusions

The identification of the existing registries constitutes an important milestone for the community of health professionals and rCTDs patients, as it allows to build on the current state of the art, while avoiding any duplication of efforts. Thanks to the creation of the TogethERN ReCONNET infrastructure, the gaps identified in this study will be addressed with a multi- stakeholder and patient-centered approach, that will on one side link all existing registries by applying the FAIR (Findable, Accessible, Interoperable and Reusable) principles and on the other hand, by creating new registries for those diseases that are not yet covered, thus contributing to create new knowledge on rCTDs and to shape a new scenario for rCTDs in Europe.

## Author contributions

MB, FD, and DM wrote the manuscript. All authors contributed in the finalization of the draft and approved the final version.

## Funding

This work was funded by the European Union's Health Program (2014–2020). ERN ReCONNET is one of the 24 European Reference Networks (ERNs) approved by the ERN Board of Member States. The ERNs are co-funded by the EC (European Commission) (grant no. 947700).

## Conflict of interest

The authors declare that the research was conducted in the absence of any commercial or financial relationships that could be construed as a potential conflict of interest.

## Publisher's note

All claims expressed in this article are solely those of the authors and do not necessarily represent those of their affiliated organizations, or those of the publisher, the editors and the reviewers. Any product that may be evaluated in this article, or claim that may be made by its manufacturer, is not guaranteed or endorsed by the publisher.

## Author disclaimer

The content of this publication represents the views of the authors only and it is their sole responsibility; it cannot be considered to reflect the views of the EC and/or the Consumers, Health, Agriculture and Food Executive Agency (CHAFEA) or any other body of the European Union. The EC and CHAFEA do not accept any responsibility for use that may be made of the information it contains.
