# Supplementary material for: An overlook on the current registries for rare and complex connective tissue diseases and the future scenario of TogethERN ReCONNET
Source: Front Med (Lausanne). 2022 Sep 26;9:889997. doi: 10.3389/fmed.2022.889997 (PMC9549150; doi:10.3389/fmed.2022.889997)
Supplement: Supplementary file 1 [file Table_1.DOCX]

|  | **N** | **Name of the registry** | **Geographic coverage** | **Starting year** | **Still ongoing** | **Disease(s) covered** | **Population/hospital based** | **Set of data collected** | **Website/link/citation/notes** |
| --- | --- | --- | --- | --- | --- | --- | --- | --- | --- |
| Antiphospholipid Syndrome | 1 | Italian Registry of Antiphospholipid Antibodies | national | 1989 | N | APS | hospital-based | laboratory, clinical | Finazzi G. The Italian Registry of Antiphospholipid Antibodies. Haematologica. 1997 Jan-Feb;82(1):101-5. PMID: 9107095. |
|  | 2 | International Pediatric APS Registry | international | 2004 | Y | Pediatric APS | hospital-based | clinical, laboratory, treatment | Avcin T, Cimaz R, Rozman B; Ped-APS Registry Collaborative Group. The Ped-APS Registry: the antiphospholipid syndrome in childhood. Lupus. 2009 Sep;18(10):894-9. doi: 10.1177/0961203309106917. PMID: 19671789. |
|  | 3 | CAPS Registry | international | 2000 | Y | CAPS | hospital-based | clinical, laboratory, treatment | Rodríguez-Pintó I et al. CAPS Registry Project Group (European Forum on Antiphospholipid Antibodies). Catastrophic antiphospholipid syndrome (CAPS): Descriptive analysis of 500 patients from the International CAPS Registry. Autoimmun Rev. 2016 Dec;15(12):1120-1124. doi: 10.1016/j.autrev.2016.09.010. Epub 2016 Sep 15. PMID: 27639837. |
|  | 4 | APS ACTION Registry | international | 2012 | Y | persistently aPL+ with or without other systemic autoimmune diseases | - | demographics, history, treatment | https://doi.org/10.1007/s11926-021-01008-8 |
|  | 5 | ARCH-APS | national (the Netherlands) | 2019 | Y | APS | hospital-based | - | www.arch.nl |
|  | 6 | COVIDAPS | European | 2020 | Y | APS and COVID-19 | hospital-based | clinical, epidemiological, laboratory, treatment | covidaps.wordpress.com |
|  | 7 | EUROAPS: European Registry on Obstetric APS | international | 2011 | Y | APS | hospital-based | clinical, epidemiological, laboratory, treatment | euroaps.wordpress.com |
|  | 8 | REGAS | National (Spain) | 2015 | Y | APS | hospital-based | Clinical, immunological |  |
|  | 9 | ENROL: European Rare Blood Disorders Platform | Multicentric (Europe) | 2020 | Y | APL | hospital-based | epidemiological, clinical, prognosis, treatment | http://www.eurobloodnet.com/enrol/ |
| Ehlers-Danlos | 10 | Nordic Database for Rare Diseases | national (Denmark) | 2007 | Y | EDS | hospital-based | epidemiological | https://raredis.eu/front-page/#forskning |
|  | 11 | Norwegian registry on rare disorders | national | 2017 | Y | EDS | population-based | epidemiological | https://sjeldenregisteret.no/ |
|  | 12 | The Finnish Register of Visual Impairment, Näkövammarekisteri | national (Finland) | - | Y | EDS (visual impairment) | population-based | epidemiological | https://thl.fi/fi/tilastot-ja-data/aineistot-ja-palvelut/rekisterien-tietosuojailmoitukset/nakovammarekisteri |
|  | 13 | ERN [EYE] - REDgistry: An interoperable sustainable European Rare Eye Disease Registry | european | appears to be yet to begin | - | EDS (visual impairment) | hospital-based | epidemiological, clinical, treatment | https://webgate.ec.europa.eu/chafea_pdb/health/projects/947444/summary |
|  | 14 | RetDis Database: blood or DNA samples of patients and families with inherited eye diseases | european | 1992 | Y | EDS (inherited eye diseases) | population-based | biobank | http://www.eye-tuebingen.de/wissingerlab/ |
|  | 15 | RetDis Database: clinical descriptions of patients and families with inherited eye diseases | international | - | N | EDS (inherited eye diseases) | population-based | clinical, epidemiological | http://www.eye-tuebingen.de/wissingerlab/ |
|  | 16 | ERN [ITHACA] - ILIAD Rare Diseases patient registry: an International Library of Intellectual disability and Anomalies of Development | european | yet to begin |  | EDS | hospital-based | clinical, epidemiological | https://ern-ithaca.eu/new-orphanet-summaries-updated-by-ern-ithaca-experts/ |
|  | 17 | UK10K RARE NEUROMUSCULAR - Genetic neuromuscular diseases variant database | international | - | Y | EDS (myopathic) | population-based | genome biobank | https://ega-archive.org/studies/EGAS00001000101 |
|  | 18 | Human DNA and cell biobank of Genethon - genetic diseases, mainly neuromuscular diseases - part of the EuroBioBank network | european | 1991 | Y | EDS (myopathic) | population-based | biobank | https://www.genethon.fr/en/rd-2/dna-and-cell-bank/ |
|  | 19 | Neuromuscular Tissue Bank (NMTB) (EuroBioBank partner) | european | 2001 | Y | EDS (myopathic) | population-based | biobank | http://www.eurobiobank.org/about/history/ |
|  | 20 | NMD-ES: Spanish Registry of Neuromuscular Diseases | national | - | Y | EDS (myopathic) | hospital-based | epidemiological, clinic | http://www.neuromuscularbcn.org/registros/registro-espanol-de-enfermedades-neuromusculares-nmd-es/ |
|  | 21 | Biobank Intellectual Disability and Multiple Congenital Anomalies (DNA) | regional (the Netherlands) | - | Y | EDS | population-based | biobank, epidemiological | https://www.orpha.net/data/prj/NL/ID126065NL.pdf |
|  | 22 | CMDIR: congenital muscular dystrophy international registry | international | - | - | EDS | population-based | epidemiological, clinical | http://www.cmdir.org/index.php?lang=fr |
|  | 23 | EDS & HSD Global Registry & Repository | international | - | Y | EDS, hypermobility spectrum disorders | population-based (patients can register themselves) | Epidemiological, clinical | https://www.ehlers-danlos.com/eds-global-registry/ OR https://www.ehlers-danlos.com/global-registry-and-repository-launch/ |
|  | 24 | RaDiCo registry for vascular EDS in France | National (France) | - | Y | EDS | - | - | https://www.radico.fr/fr/connaitre-radico/nos-cohortes-et-autres-programmes-associes/ 80-radico/155-radico-sed-vasc |
|  | 25 | Registry of Ehlers-Danlos Syndrome (RED) | - | 2014 | Y | EDS | Hospital-based (enrolling by invitation) | epidemiological, clinical, genetics, treatment | http://www.ior.it/en/curarsi-al-rizzoli/registries-rare-hereditary-diseases-registries-details AND https://clinicaltrials.gov/ct2/show/NCT04133272?cond=Ehlers-Danlos+Syndrome&draw=2 |
|  | 26 | VASCERN Registry | - | 2020 | Y | EDS | - | - | https://vascern.eu/expertise/rare-diseases-wgs/medium-size-arteries-wg/ |
| IgG4-related disease | 27 | National registry for retroperitoneal fibrosis | national (Germany) | - | Y | IgG4-RD | population-based | epidemiological, clinical | https://www.helios-gesundheit.de/kliniken/wuppertal/unser-angebot/unsere-fachbereiche/urologie-und-kinderurologie-prostatazentrum/morbus-ormond/ |
|  | 28 | French multicentre database for IgG4-RD | national (France) | 2009-2016 | N | IgG4-RD | - | clinical, radiological, treatment | Ebbo M, et al. Long-term efficacy and safety of rituximab in IgG4-related disease: Data from a French nationwide study of thirty-three patients. PLoS One. 2017 Sep 15;12(9):e0183844. doi: 10.1371/journal.pone.0183844. PMID: 28915275; PMCID: PMC5600376. |
|  | 29 | IgG4-RD Spanish Register | National (12 spanish centers) | - | Y | IgG4-RD | Hospital-based |  |  |
|  | 30 | IgG4-RD Registry Study | European (19 centers) | 2010 | Y | IgG4-RD | Hospital-based | clinical, epidemiological, treatment | https://igg4-rd.ndm.ox.ac.uk/igg4-rd-registry-study |
|  | 31 | Registre Français IgG4 | National (France) | 2019 | Y | IgG4-RD | - | - |  |
| Idiopathic inflammatory Myopathies | 32 | MYOGEN international genetics consortium in myositis | international | - | - | Adult and juvenile DM | population-based | biobank |  |
|  | 33 | JDCBS - JDM Cohort Biomarker Study and Repository (formally known as the National Registry and Repository) | UK | 2000 | Y | JDM | population-based | clinical, epidemiological | https://juveniledermatomyositis.org.uk/ |
|  | 34 | Electronic myositis register (EUROMYOSITIS) | European (+3 centers from China, Japan, US) | 2010 | Y | Adult and Juvenile IIM | Hospital-based (each hospital only has access to their own data) | clinical, epidemiological | (https://euromyositis.eu) |
|  | 35 | Network Myonet | global | - | Y | IIM | Hospital-based (each hospital only has access to their own data) | clinical, epidemiological | (http://www.myonet.eu) |
|  | 36 | Registry of Inflammatory Myopathies in the autonomous community of Madrid (REMICAM) | multicenter, regional, Madrid | - | N | IIM | hospital-based | clinical, epidemiological |  |
|  | 37 | The Childhood Arthritis and Rheumatology Research Alliance (CARRA) Legacy Registry | international (mainly US and Canada; one location in Italy and Israel) | 2002 | Y | JDM | population-based | clinical, epidemiological | (https://www.carragroup.org) |
|  | 38 | International Myositis Assessment and Clinical Studies Group (IMACS) Outcomes Data Repository | international | - | Y | IIM | population-based | clinical, epidemiological | (http://www.niehs.nih.gov/research/resources/imacs/researchguidelines/index.cfm) |
|  | 39 | The MYOVISION registry | regional, USA | - (ended by 2019) | N | IIM | hospital-based | clinical, epidemiological | https://www.myositis.org/blog/myovision-official-notice/ |
|  | 40 | Hopkins Myositis Research Database | USA, single center | - | Y | IIM | hospital-based | clinical, epidemiological, biobank | https://www.hopkinsmyositis.org/research/ |
|  | 41 | UKMYONET | Multicenter, United Kingdom | - | N | IIM | population-based | clinical, epidemiological | Became part of euro myositis and myonet |
|  | 42 | Hungarian Myositis Workgroup | Multicenter, Hungary | - | - | IIM | hospital-based | clinical, epidemiological |  |
|  | 43 | SweMyoNet | National, Sweden | - | Y | IIM | population-based | clinical, epidemiological | (http://srq.nu/) |
|  | 44 | REGAS (Registry of Antisynthetase Syndrome) | Multicenter, Spain | - | - | Antisynthetase syndrome |  | clinical, epidemiological |  |
|  | 45 | CHUM Myositis Registry | Multicenter, Canada | - | - | IIM | hospital-based | clinical, epidemiological |  |
|  | 46 | UK National Neuromuscular Database for Personalised Medicine | UK | - | Y | IIM | population-based | clinical, epidemiological | https://www.ucl.ac.uk/centre-for-neuromuscular-diseases/research/research-core-activities/uk-national-neuromuscular-database-personalised-medicine |
|  | 47 | - | National (France) | - | Y | IIM | Population-based | Clinical, epidemiological |  |
|  | 48 | AENEAS | international | 2017 | Y | Antisynthetase syndrome | Hospital-based | Clinical, epidemiological, immunological | https://www.researchgate.net/publication/312327900_AENEAS_American_European_NEtwork_of_Antisynthetase_Syndrome_collaborative_group_Clinical_spectrum_time_course_in_anti_Jo-1_positive_anti-synthetase_syndrome_Results_from_an_international_retrospective |
|  | 49 | CLASS | international | 2019 | Y | Antisynthetase syndrome | Hospital-based | Clinical, epidemiological, immunological | https://www.eular.org/recommendations_eular_acr.cfm |
|  | 50 | GEM (Ghent Early Myositis cohort) | Regional (Ghent) | 2019 | Y | IIM | Hospital-based | Clinical, epidemiological, treatment | https://studiesredcap.uzgent.be/ |
|  | 51 | MyoDan | National (Denmark) | 2015 | Y | IIM | Hospital-based | clinical | www.myodan.danbio-online.dk |
|  | 52 | MyositEst | International | 2011 | Y | IIM | Hospital-based | clinical |  |
|  | 53 | Myositis classification Paris | Regional (Paris) | - | N | IIM | Hospital-based | clinical | https://www.institut-myologie.org/en/2018/09/20/myositis-a-new-classification-system-based-on-phenotypic-biological-and-immunological-criteria/ |
|  | 54 | PREMIA | Regional (eastern France) | 2006 | N | IIM | Population-based | clinical, epidemiological |  |
|  | 55 | MEDRAS | National (Spain) | 2019 | Y | Clinically Amyopathic Dermatomyositis | Hospital-based | clinical, immunological |  |
|  | 56 | The Inclusion Body Myositis Disease Registry at Yale | International | - | Y | Inclusion body myositis | Hospital-based | Demographics, clinical, | https://ysph.yale.edu/ibmregistry/ |
|  | 57 | PANLAR Myositis Registry | American | - | Y | IIM | Hospital-based | clinical, epidemiological | https://www.panlar.org/ |
|  | 58 | CIMS | Canada | - | Y | IIM | - | - | http://craj.ca/archives/2019/English/Spring/pdf/CRAJ_Spring_2019_CIMS.pdf |
|  | 59 | Brazilian registry of juvenile dermatomyositis | multicenter in Brazil | - | N | IIM | hospital-based | clinical, epidemiological | https://www.repositorio.unifesp.br/handle/11600/43323 |
| Systemic Lupus Erythematosus | 60 | Core documentation of children and adolescents with rheumatic diseases | national (Germany) | 1997 | Y | Pediatric and neonatal SLE | hospital-based | clinical, epidemiological | https://tinyurl.com/yf8mndmd |
|  | 61 | Offspring of SLE Mothers Registry (OSLER) | - | 1989 | N | SLE | hospital-based | clinical, epidemiological | Vinet É et al. Brief Report: Causes of Stillbirths in Women With Systemic Lupus Erythematosus. Arthritis Rheumatol. 2016 Oct;68(10):2487-91. doi: 10.1002/art.39742. PMID: 27159385. |
|  | 62 | RELESSER (Spanish Society of Rheumatology Lupus Registry) | national | 2013 | Y | SLE | hospital-based | clinical, epidemiological, treatment | Rúa-Figueroa I et al. National registry of patients with systemic lupus erythematosus of the Spanish Society of Rheumatology: objectives and methodology. Reumatol Clin. 2014 Jan-Feb;10(1):17-24. English, Spanish. doi: 10.1016/j.reuma.2013.04.013. Epub 2013 Jul 17. PMID: 23871155. |
|  | 63 | Lupus Family Registry and Repository | national (USA) | 1990’s | Y | SLE | hospital-based | clinical, epidemiological, biobank, genetics | Rasmussen A et al. The lupus family registry and repository. Rheumatology (Oxford). 2011 Jan;50(1):47-59. doi: 10.1093/rheumatology/keq302. Epub 2010 Sep 23. PMID: 20864496; PMCID: PMC3307518. |
|  | 64 | International Registry for Biologics in SLE (IRBIS) | international | 2013 | Y | SLE | hospital-based | clinical, epidemiological, treatment | Adamichou C, Flouri I, Fanouriakis A, et al. Development and Implementation of a Pilot Registry for Monitoring the Efficacy and Safety of Novel Therapies in Patients with Systemic Lupus Erythematosus. *Mediterr J Rheumatol*. 2020;31(1):87-91. Published 2020 Mar 31. doi:10.31138/mjr.31.1.87 |
|  | 65 | Franco-Canadian program for the surveillance and pharmacoepidemiological evaluation of risk factors for rare diseases (PGRx): Lupus | international | 2008 | Y | SLE (pediatric and adult) | population-based | pharmaco-surveillance, clinical | https://epidemiologie-france.aviesan.fr/epidemiology/records/pgrx-lupus/eng-gb |
|  | 66 | The Cretan Community-Based Lupus Registry | national | 2012 | - | SLE | hospital-based | clinical, epidemiological | Gergianaki I et al. Epidemiology and burden of systemic lupus erythematosus in a Southern European population: data from the community-based lupus registry of Crete, Greece*Annals of the Rheumatic Diseases* 2017;**76:**1992-2000. |
|  | 67 | DAIRE | National (the Netherlands) | - | Y | SLE | Hospital-based | Clinical, epidemiological, treatment |  |
|  | 68 | LIRE | National (Italy) | - | - | SLE | Population-based |  |  |
|  | 69 | Lula | German | 2005 | Y | SLE | - |  |  |
|  | 70 | Romanian SLE Registry | National (Romania) | 2019 | Y | SLE | Hospital-based  (based on bDMARD prescription - belimumab) | Clinical, particular focus on treatment |  |
|  | 71 | SLEGEIST | Denmark | 2018 | Y | SLE | - | clinical |  |
|  | 72 | SLICC | International | - | Y | SLE | Hospital-based | clinical, epidemiological | https://sliccgroup.org |
|  | 73 | Swiss SLE Cohort Study | national (Switzerland) |  | Y | SLE | hospital-based | clinical, epidemiological |  |
|  | 74 | SICCA International Registry - Sjögren’s International Collaborative Clinical Alliance | international (European and American countries) | 2004 | N | SS | population-based | clinical, epidemiological, biobank | https://sicca-online.ucsf.edu/ |
|  | 75 | The Big Data Sjögren Consortium | international | 2014 | Y | SS | population-based | clinical, epidemiological | https://pubmed.ncbi.nlm.nih.gov/31464669/ |
|  | 76 | SJÖGREN'S-SER | National (Spain) | 2012 | Y | SS | Hospital-based | epidemiological, clinical, serological characteristics, treatments and complications | https://sjogren.ser.es/ |
|  | 77 | UK Primary Sjogren's Syndrome Registry (UKPSSR) | UK | - | Y | SS | population-based | clinical, epidemiological | http://www.sjogrensregistry.org/index.php http://www.sjogrensregistry.org/registry/ |
|  | 78 | GEAS-SS REGISTRY | Spain | 2005 | - | SS | population-based | clinical, epidemiological |  |
|  | 79 | ASSESS | France | - | Y | SS | Hospital-based | clinical, epidemiological | https://www.fai2r.org/protocoles-de-recherche-clinique-en-cours/gougerot-sjogren-protocoles-cours/ |
|  | 80 | Romanian SS registry | Regional | 2019 | Y | SS | Hospital-based | Demographic, clinical, laboratory, ultrasound data |  |
|  | 81 | Sjogren’s registry | National (Italy) | - | - | SS | Hospital-based | - |  |
|  | 82 | Swiss Sjögren Registry | National (Switzerland) | 2021 | Y | SS | Hospital-based | Clinical, epidemiological, biobank |  |
|  | 83 | BESSTT (Belgian Sjögren Syndrome Transition Trial) | Local (Ghent) | 2019 | Y | SS | hospital-based | - | Deroo L, et al. Economic burden in Sjögren’s syndrome. Abstract on the Belgian Congress on Rheumatology 2021. |
| Systemic Sclerosis | 84 | Systemic sclerosis Progression INvestiGation (SPRING) | National (Italy) | 2018 | Y | SSc, Raynaud's phenomenon, VEDOSS | population-based | clinical, epidemiological | https://www.reumatologia.it/en/cmsx.asp?IDPg=395 |
|  | 85 | Spanish Scleroderma Registry (RESCLE) | Spanish - Departments of Internal Medicine of 29 hospitals nationwide | 2006 | N | SSc | hospital-based | clinical, epidemiological, laboratory, capillaroscopical data | https://www.fesemi.org/grupos/autoinmunes/investigacion/registros/registro-espanol-de-pacientes-con-esclerodermia-registro-rescle |
|  | 86 | EULAR Scleroderma Trials & Research (EUSTAR) | european | 2004 | Y | SSc | population-based | clinical, epidemiological, biobank | https://www.eustar-online.org |
|  | 87 | GEPRO - the Pronuclear Project SSc Study Group | Brazil - 28 university centers from different geographical areas all over Brazil | 2003 | N | SSc | population-based- | clinical, epidemiological, immunologic |  |
|  | 88 | Collaborative National Quality and Efficacy  Registry (CONQUER) for Scleroderma | United States | 2018 | Y | SSc | population-based | clinical, epidemiological | https://srfcure.org/research/conquer/ |
|  | 89 | Prospective Registry of Early Systemic Sclerosis (PRESS) | USA | 2011-2017 | N | SSc | population-based | clinical, epidemiological | (became CONQUER - above) |
|  | 90 | GUSU (Ghent University Scleroderma Unit) / BSSC (Belgian Systemic Sclerosis Cohort) | National (Belgium) | 2006 | Y | SSc | Hospital-based | Clinical, epidemiological | https://studiesredcap.uzgent.be/ www.uzgent.be/systeemsclerose |
|  | 91 | Register of patients with scleroderma | National (France) | 2015 | Y | SSc | Hospital-based | Clinical, epidemiological |  |
|  | 92 | SCLERODAN | Denmark | 2021 | Y | SSc | Hospital-based | Clinical, epidemiological |  |
|  | 93 | Scleroderma Patient-centered Intervention Network (SPIN) Hand Program | International | 2018 | Y | SSc | Hospital-based | Clinical, epidemiological |  |
|  | 94 | The Genome Research in African American Scleroderma Patients (GRASP) Project | United States- 23 participating US academic centers | - | Y | SSc | population-based | clinical, epidemiological, biobank | https://srfcure.org/research/grasp/ |
|  | 95 | UK scleroderma cohort (SMART) | UK, regional | - | - | SSc | population-based | clinical, epidemiological, biobank | https://www.hra.nhs.uk/planning-and-improving-research/application-summaries/research-summaries/uk-smart-database-and-tissue-bank-version-4-16012015/ |
|  | 96 | The University of Pittsburgh Scleroderma Databank | regional - Pittsburgh | 1980 | Y | SSc | hospital-based | clinical, epidemiological, biobank | https://dom.pitt.edu/rheum/centers-institutes/scleroderma/systemicsclerosiscenter/database/ |
|  | 97 | Australian Scleroderma Cohort Study (ASCS) from the Australian Scleroderma Interest Group (ASIG) | Australian | 2007 | Y | SSc | population-based | clinical, epidemiological | https://rheumatology.org.au/For-Healthcare-Professionals/Special-Interest-Groups/Australian-Scleroderma-Interest-Group |
|  | 98 | The Canadian Scleroderma Research Group CSRG registry | Canada | 2004 | Y | SSc | population-based | clinical, epidemiological | http://www.canadiansclerodermaresearchgroup.org/ |
|  | 99 | The Digital Ulcers Outcome (DUO) Registry | european | - | Y | DU/SSc | population-based | clinical, epidemiological |  |
|  | 100 | South Australian Scleroderma Register (SASR) | Australian - regional | 1993 | - | SSc | hospital-based | clinical, epidemiological |  |
|  | 101 | Pulmonary Hypertension Assessment and Recognition of Outcomes in Scleroderma (PHAROS) | International multicenter study conducted at 18 US and Canadian sites | 2010 | - | subjects with SSc at high risk for or with incident pulmonary hypertension (PH) | hospital-based | clinical, epidemiological, laboratory | Hinchcliff M et al. PHAROS Investigators. Pulmonary Hypertension Assessment and Recognition of Outcomes in Scleroderma (PHAROS): baseline characteristics and description of study population. J Rheumatol. 2011 Oct;38(10):2172-9. doi: 10.3899/jrheum.101243. Epub 2011 Aug 15. PMID: 21844142; PMCID: PMC3230328. |
|  | 102 | Morphea in Adults and Children (MAC) | United States | 2007 | Y | both children and adults with morphea | population-based | clinical, epidemiological | https://www.utsouthwestern.edu/education/medical-school/departments/dermatology/research/morphea-registry/ |
| Multiple-disease registries | 103 | WAPS study registry | international (Italy, Norway, Poland, Argentina, and Czech Republic) | 1997 | N | APS, SLE | hospital-based | epidemiological, clinical, laboratory, biobank | Finazzi G et al. A randomized clinical trial of high-intensity warfarin vs. conventional antithrombotic therapy for the prevention of recurrent thrombosis in patients with the antiphospholipid syndrome (WAPS). J Thromb Haemost. 2005 May;3(5):848-53. doi: 10.1111/j.1538-7836.2005.01340.x. PMID: 15869575. |
|  | 104 | ACR’s RISE registry | national | 2014 | Y | SLE, SSc, IIM, UCTD, RP, SS | hospital-based | clinical, epidemiological | Yazdany J et al. Rheumatology Informatics System for Effectiveness: A National Informatics-Enabled Registry for Quality Improvement. Arthritis Care Res (Hoboken). 2016 Dec;68(12):1866-1873. doi: 10.1002/acr.23089. PMID: 27696755; PMCID: PMC5125872. |
|  | 105 | GRAID1/GRAID2 registry | national (Germany) | 2010 | N | SLE on RTX/SLE, SSc, SS, IIM on biologic off-label therapy | - | clinical, epidemiological, treatment | Witt M, Grunke M et al. German Registry of Autoimmune Diseases (GRAID) Investigators. Clinical outcomes and safety of rituximab treatment for patients with systemic lupus erythematosus (SLE) - results from a nationwide cohort in Germany (GRAID). Lupus. 2013 Oct;22(11):1142-9. doi: 10.1177/0961203313503912. PMID: 24057058. |
|  | 106 | Swiss Rare Disease Registry (SRDR) | national (Switzerland) | 2014 | Y | including all rCTDs and EDS | population-based adults & children | clinical, epidemiological | https://www.fmh.ch/themen/qualitaet-saqm/register/medizinische-register.cfm?fuseaction_sea=detail&id=71#a1 |
|  | 107 | SIERrm: Information System on rare diseases in the Region of Murcia (Spain) | regional (Murcia-Spain) | 2015 | Y | including SLE, APL, IgG4, EDS | population-based adults & children | clinical, epidemiological | http://www.murciasalud.es//pagina.php?id=167837&idsec=1084 |
|  | 108 | ReeR: Spanish National Registry of Rare Diseases (SpainRDR) | national | 2015 | Y | including SLE, APL, IgG4, EDS | population-based adults & children | epidemiological | https://registroraras.isciii.es/Comun/Inicio.aspx |
|  | 109 | Registry for rare diseases in Extremadura | regional (Spain) | 2015 | Y | including SLE, APL, IgG4, EDS | population-  based adults & children | epidemiological | https://saludextremadura.ses.es/web/enlaces?tag=enfermedades%20raras&refMenu=382 |
|  | 110 | SIER-CV: Information System on rare diseases in Valencian Community | regional | 1999 | Y | including SLE, APL, IgG4, EDS | population-based | epidemiological | http://www.sp.san.gva.es/ |
|  | 111 | Population registry of rare diseases and congenital anomalies of Cantabria | regional | 2013 | Y | including SLE, APL, IgG4, EDS | population-based | epidemiological | http://boc.cantabria.es/boces/verAnuncioAction.do?idAnuBlob=242983 |
|  | 112 | Population registry of rare diseases of Balearic Islands | regional | - | N | including SLE, APL, IgG4, EDS | population-based | epidemiological | - |
|  | 113 | Rare disease registry of Aragon | regional | 2013 | Y | including SLE, APL, IgG4, EDS | population-based | epidemiological | http://www.boa.aragon.es/cgi-bin/EBOA/BRSCGI?CMD=VERDOC&BASE=BZHT&PIECE=BOLE&DOCR=6&SEC=FIRMA&RNG=10&SEPARADOR=&&PUBL-C=20130315 |
|  | 114 | Registry for rare diseases in Andalusia | regional | 2013 | Y | including SLE, APL, IgG4, EDS |  |  |  |
|  | 115 | French National Registry for Rare Diseases (BNDMR) | national | - | Y | including SLE, APL, IgG4, EDS | hospital-based | epidemiological | https://www.bndmr.fr/ |
|  | 116 | Central Registry of Rare Diseases (CRRD) | national | 2014 | Y | including SLE, APL, IgG4, EDS | hospital-based | epidemiological | https://rarediseases.sciensano.be/en/registry |
|  | 117 | The Finnish Hematology Registry and Clinical Biobank (FHRB Biobank) | national | 2013 | Y | including APL | hospital-based population-based | biobank, epidemiological | https://www.fhrb.fi/fhrb/background-and-operating-principles.html |
|  | 118 | Austrian Country Node of the Human Variome Project (HVP) | national | - | - | including EDS | - | genome variants database | http://www.oegh.at/index.php?option=com_content&view=article&id=36&Itemid=25 |
|  | 119 | Korean Mutation Database for Rare Diseases | national | - | Y | including EDS | - | genome variants database | https://kmd.nih.go.kr/kmd/kmd?type=emain |
|  | 120 | Biobank of the Estonian Genome Centre | national | - | Y | including EDS | population-based | Epidemiological, biobank | https://www.geenivaramu.ee/en |
|  | 121 | Basque Biobank | national | 2012 | Y | including EDS | hospital-based | biobank | http://www.biobancovasco.org/ |
|  | 122 | BioNER: Biobank of the Institute of Rare Diseases Research/Institute of Health Carlos III | national (Spain) | 2012 | Y | including EDS | - | biobank | http://bioner.isciii.es/ |
|  | 123 | CIBERER Biobank (Biobank of rare diseases) | regional (valencia) | 2007 | Y | including EDS | population-based | biobank, epidemiological | http://www.ciberer-biobank.es/ |
|  | 124 | Biobanca di linee cellulari e DNA da pazienti affetti da malattie genetiche | national (Italy) | 70’s | N | including EDS | population-based | biobank | http://dppm.gaslini.org/biobank/ |
|  | 125 | Danish National Patient Registry (DNPR) | Denmark | 1977 | Y | including rCTDs | population-based | clinical, epidemiological |  |
|  | 126 | ARCH | National (the Netherlands) | 2019 | Y | SLE, SSc, Sjogren (and ANCA vasculitis) | Hospital-based | Clinical, epidemiological |  |
|  | 127 | Atrioventricular block register | National (France) | 2014 | Y | SLE, SS | Population-based | - |  |
|  | 128 | CCISS | Regional (the Netherlands) | 2009 | Y | SSc, UCTD, MCTD | Hospital-based | Clinical, epidemiological, treatment |  |
|  | 129 | DanBio | Denmark | - | Y | multiple inflammatory diseases | - |  | https://danbio-online.dk/front-page |
|  | 130 | DNSS | German | 2003 | Y | SSc, MCTD, UCTD | Population-based | Clinical, epidemiological | www.sklerodermie.info |
|  | 131 | Italian National Register of Rare Diseases | National | 2001 | Y | APL, EDS, IIM, MCTD, RP, SSc, UCTD | Population-based | Clinical, epidemiological | https://www.iss.it |
|  | 132 | Kerndokumentation | German | 2003 | Y | all inflammatory rheumatic diseases | - | Clinical, epidemiological |  |
|  | 133 | RARENET | European | - | N | pSS, IIM, SLE, vasculitis, APS | Hospital-based | - | https://www.rarenet.eu |
|  | 134 | Register of Patients with Anti-Phospholipids Syndrome (APS) and/or Systemic Lupus Erythematosus (SLE) | National | 2017 | Y | APS, SLE | Hospital-based | Clinical, epidemiological, treatment |  |
|  | 135 | TATA | France | - | Y | multiple inflammatory diseases | - | Clinical, epidemiological |  |
|  | 136 | The Registry to Evaluate Early and Long-term PAH Disease Management (REVEAL Registry) | United States- 55-center observational US | - | - | PAH patients (some due to rCTDs) | population-based | clinical, epidemiological, treatment | https://pubmed.ncbi.nlm.nih.gov/26066077/ |
|  | 137 | Indian Health Service Lupus Registry Project | 3 US regions | 2007 | - | SLE, MCTD | population-based | clinical, epidemiological | https://www.cdc.gov/lupus/funded/lupus-studies.htm |
|  | 138 | French PH Registry | France (French reference center for severe PH and 23 regional competence centers) | - | - | PAH (some due to rCTDs) | population-based | clinical, epidemiological |  |
|  | 139 | The Registry of Pulmonary Hypertension Associated with Rheumatic Disease (REOPARD) | Korea | - | - | PAH due to rCTDs | population-based | clinical, epidemiological | http://www.korpah.or.kr/ |
|  | 140 | Rheumatic Diseases Portuguese Register (Reuma.pt) | Portugal | 2008 | Y | All rheumatic diseases | hospital-based | clinical, epidemiological | www.reuma.pt |

**Table 1**: Rare and complex connective tissue diseases registries
